# Vitamin D and allergic airway disease shape the murine lung microbiome in a sex-specific manner

**DOI:** 10.1186/s12931-016-0435-3

**Published:** 2016-09-21

**Authors:** Michael Roggenbuck, Denise Anderson, Kenneth Klingenberg Barfod, Martin Feelisch, Sian Geldenhuys, Søren J. Sørensen, Clare E. Weeden, Prue H. Hart, Shelley Gorman

**Affiliations:** 1 Section of Microbiology, Department of Biology, University of Copenhagen, Copenhagen, Denmark; 2 Telethon Kids Institute, University of Western Australia, 100 Roberts Rd, Subiaco, WA 6008 Australia; 3 The National Research Centre for the Working Environment, Copenhagen, Denmark; 4 Clinical and Experimental Sciences, Faculty of Medicine, University of Southampton, Southampton General Hospital, Southampton, UK

**Keywords:** Vitamin D, Lung, Allergic airway disease, Inflammation, Microbiome, Sex differences, *Acinetobacter*

## Abstract

**Background:**

Vitamin D is under scrutiny as a potential regulator of the development of respiratory diseases characterised by chronic lung inflammation, including asthma and chronic obstructive pulmonary disease. It has anti-inflammatory effects; however, knowledge around the relationship between dietary vitamin D, inflammation and the microbiome in the lungs is limited. In our previous studies, we observed more inflammatory cells in the bronchoalveolar lavage fluid and increased bacterial load in the lungs of vitamin D-deficient male mice with allergic airway disease, suggesting that vitamin D might modulate the lung microbiome. In the current study, we examined in more depth the effects of vitamin D deficiency initiated early in life, and subsequent supplementation with dietary vitamin D on the composition of the lung microbiome and the extent of respiratory inflammation.

**Methods:**

BALB/c dams were fed a vitamin D-supplemented or -deficient diet throughout gestation and lactation, with offspring continued on this diet post-natally. Some initially deficient offspring were fed a supplemented diet from 8 weeks of age. The lungs of naïve adult male and female offspring were compared prior to the induction of allergic airway disease. In further experiments, offspring were sensitised and boosted with the experimental allergen, ovalbumin (OVA), and T helper type 2-skewing adjuvant, aluminium hydroxide, followed by a single respiratory challenge with OVA.

**Results:**

In mice fed a vitamin D-containing diet throughout life, a sex difference in the lung microbial community was observed, with increased levels of an *Acinetobacter* operational taxonomic unit (OTU) in female lungs compared to male lungs. This effect was not observed in vitamin D-deficient mice or initially deficient mice supplemented with vitamin D from early adulthood. In addition, serum 25-hydroxyvitamin D levels inversely correlated with total bacterial OTUs, and *Pseudomonas* OTUs in the lungs. Increased levels of the antimicrobial murine ß-defensin-2 were detected in the bronchoalveolar lavage fluid of male and female mice fed a vitamin D-containing diet. The induction of OVA-induced allergic airway disease itself had a profound affect on the OTUs identified in the lung microbiome, which was accompanied by substantially more respiratory inflammation than that induced by vitamin D deficiency alone.

**Conclusion:**

These data support the notion that maintaining sufficient vitamin D is necessary for optimal lung health, and that vitamin D may modulate the lung microbiome in a sex-specific fashion. Furthermore, our data suggest that the magnitude of the pro-inflammatory and microbiome-modifying effects of vitamin D deficiency were substantially less than that of allergic airway disease, and that there is an important interplay between respiratory inflammation and the lung microbiome.

## Background

Vitamin D has been proposed as an important regulator of both inflammation [1] and the microbiome [2, 3]. Indeed, it is hypothesised that there is a protective role for vitamin D in regulating the gut microbiome, which may shape predisposition for the development of inflammatory autoimmune and allergic diseases [2–4]. Vitamin D is commonly acquired through skin exposure to ultraviolet B radiation found in sunlight and through the diet. Circulating 25-hydroxyvitamin D (25(OH)D) is typically used as a measure of vitamin D status, although 1,25-dihydroxyvitamin D (1,25(OH)_2_D) is the most active vitamin D metabolite.

Our understanding of the effects of vitamin D on the microbiome and its associations with reduced tissue inflammation is currently limited to the gastrointestinal tract. Colitis severity and bacterial numbers in the colons of mice were increased during vitamin D deficiency [5]. Dietary-induced vitamin D deficiency altered the composition of the fecal microbiome of C57Bl/6 mice, which had increased relative quantities of *Bacteroidetes*, *Firmicutes*, *Actinobacteria*, and *Gammaproteobacteria* [6]. These mice also had increased colonic injury in comparison to mice fed a vitamin D-sufficient diet, but exhibited signs of hypocalcemia [6]. In other studies, the colons of 21-day-old vitamin D-deficient mice were enriched for *Bacteroides*/*Prevotella* colony forming units, although this difference disappeared with age [7]. CYP27B1^−/−^ mice, which lack the expression of the 1α-hydroxylase enzyme, responsible for converting circulating 25(OH)D to active 1,25(OH)_2_D, had an increased fecal burden of *Proteobacterium* phylum (including *Helicobacteraceae* species) in a colitis model [8]. Treatment of CYP27B1^−/−^ mice with 1,25(OH)_2_D (1.25 μg/100 g diet) suppressed colitis severity and *Helicobacteraceae* numbers [8]. Vitamin D supplementation (980 IU vitamin D_3_/kg per week for 4 weeks) of 16 healthy young adults reduced the relative abundance of Gammaproteobacteria such as *Pseudomonas* spp. and *Escherichia/Shigella* spp., increasing bacterial richness in the upper gastrointestinal tract, but not in ileum, colon nor in stool samples [9]. Changes in *Bacteroides* spp. were identified in the stool samples of African American men (*n* = 115) with prediabetes and hypovitaminosis D after weekly supplementation with a vitamin D analogue (ergocalciferol; 50,000 IU) [10]. Together, these data suggest that dietary vitamin D can alter the gut microbiome, reducing the abundance of potentially pathogenic species with these changes linked to reduced gastrointestinal inflammation and injury.

Vitamin D may shape the microbiome through a number of interdependent mechanisms. Firstly, vitamin D regulates innate immune responses. Vitamin D can induce the expression of antimicrobial peptides and proteins, such as cathelicidins and ß-defensins, which are produced by monocytes, macrophages and epithelial cells in the skin and lung [1, 3]. Vitamin D-deficient mice had reduced colonic expression of the antimicrobial protein, angiogenin-4, which was associated with increased bacterial load, and tissue inflammation [5]. Such antimicrobials may directly kill microbiota or be involved in activating innate immune processes such as autophagy in macrophages, promoting the ingestion of microbes into phagolysosomes for neutralization [11]. Vitamin D may promote tolerant adaptive immune responses by modulating the gut microbiome. As highlighted above, there is evidence linking the modified gut microbiome and increased gut inflammation of CYP27B1^−/−^ mice, with fewer tolerogenic CD103+ dendritic cell in lamina propria [8]. These are cells responsible for shaping the regulatory T cell repertoire of the gut [12]. Ongoing presentation of bacterial antigens by dendritic cells may be required for tolerance towards gut microbes, with an essential role for tolerogenic dendritic cells and T cells to limit inflammation [13, 14]. Finally, vitamin D may up-keep epithelial integrity. Colonic epithelial cells from CYP27B1^−/−^ mice expressed reduced levels of the cell-to-cell adhesion protein, E-cadherin [8]. Assa et al. [6] demonstrated that vitamin D-deficient mice had reduced colonic epithelial barrier function, with increased permeability that associated with increased proinflammatory cytokine expression, signs of colitis and relative quantities of *Bacteroidetes, Firmicutes*, *Actinobacteria* and *Gammaproteobacteria* in the intestinal microbiome.

In healthy human lungs, the bronchial tree holds approximately 2000 distinct bacterial genomes per cm^2^ [15]. There are diverse microbial communities, with *Bacteroidetes*, *Proteobacteria* and *Firmicutes* the mostly commonly identified at the phylum level [16]. Similarly, the healthy mouse lung microbiome is dominated by species of these phyla and also *Actinobacteria* and *Cyanobacteria* [17]. The lung microbiome changes from infancy through to adulthood, with the ratio of *Bacteroidetes:Firmicutes/Gammproteobacteria* increasing with age in specific-pathogen-free mice [18]. However, dysregulation of the microbiome of the lungs and associated tissues could contribute towards respiratory inflammation.

Indeed, microbial communities shift in the respiratory tract during respiratory disease [15, 19–23]. Hilty et al. [15] demonstrated that pathogenic proteobacteria were more common in bronchial brushings of the left upper lung lobe of adult asthmatics (*n* = 11) or patients with chronic obstructive pulmonary disease (COPD) (*n* = 5) than controls (*n* = 8), with similar findings in the bronchoalveolar lavage fluid (BALF) of asthmatic children (*n* = 13). Lung tissue samples from patients with COPD (*n* = 8) had increased bacteria of the *Lactobacillus* genus with bacterial communities distinct to healthy controls (*n* = 8), smokers without COPD (*n* = 8) or patients with cystic fibrosis (*n* = 8) [20]. *Lactobacillus*, *Pseudomonas*, and *Rickettsia* species were enriched in endobronchial brush samples from patients with chronic persistent asthma (*n* = 39) in comparison to healthy controls (*n* = 19) [21]. The bronchial brushings of patients with severe asthma (*n* = 30) were enriched with *Actinobacteria* and a member of the *Klebsiella* genus in comparison to healthy controls (*n* = 8) and mild-to-moderate asthmatics (*n* = 41) [22]. Together, these findings suggest that pathogenic bacteria are more common in the lungs and associated respiratory tissue of patients with respiratory disease, which may vary depending on the site examined.

Animal models offer the opportunity of dissecting various factors that change the lung microbiome to initiate and/or exacerbate inflammatory or allergic lung diseases. The effect of vitamin D deficiency on the lung microbiome has not been specifically investigated in vivo. We previously observed that inflammatory cell numbers in the BALF of vitamin D-deficient male mice with allergic airway disease were associated with increased bacterial load in the lungs [24]. Low levels of circulating 25(OH)D were associated with increased numbers of eosinophils and neutrophils in BALF in male mice with allergic airway disease [24]. Increased levels of BALF inflammation and microbial load in the lungs of initially vitamin D-deficient mice were reversed by dietary vitamin D supplementation [24]. In the current study, we hypothesised that vitamin D deficiency would alter the lung microbiome of naïve mice, potentially contributing towards increased respiratory inflammation, prior to the initiation of allergic airway disease.

## Methods

### Mice and diets

**Fig. 1:**
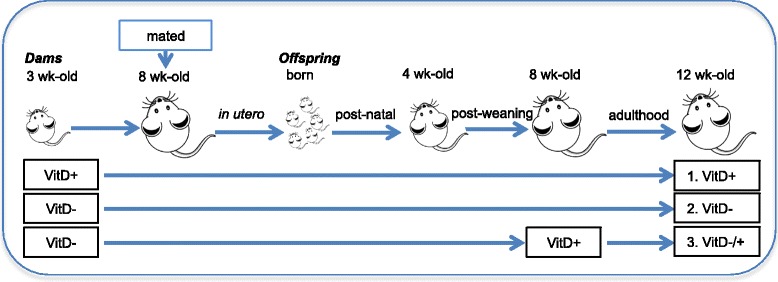
The dietary intake of mice in each treatment. In (A), female BALB/c mice (dams) were fed vitamin D-containing (VitD+) or vitamin D-null (VitD−) diets from 3 weeks of age and used to produce offspring. A subgroup of the vitamin D-deficient offspring was fed a vitamin D-supplemented diet from 8 weeks of age (VitD−/+)

All experiments were performed according to the ethical guidelines of the National Health and Medical Research Council of Australia and with approval from the Telethon Kids Institute Animal Ethics Committee (AEC#238). Mice were purchased from the Animal Resources Centre, Western Australia. Mice were maintained under specific-pathogen-free conditions. Female 3 week-old BALB/c mice were fed semi-pure diets, which were either supplemented with vitamin D_3_ (2,280 IU vitamin D_3_/kg, SF05-34, Specialty Feeds, Perth, Western Australia) or not (0 IU vitamin D_3_/kg, SF05-033, Specialty Feeds) as previously described [24, 25]. From 8 weeks of age, female mice were mated with male BALB/c mice for up to 2 weeks. These male mice were fed standard mouse chow until breeding (Specialty Feeds, containing 2,000 IU vitamin D_3_/kg). Litter sizes from dams fed either diet were a mean of 5 pups per litter, with equal proportions of females and males. Offspring born were fed the vitamin D-replete or -deficient diets for the rest of the experiment, except in experiments when initially vitamin D-deficient mice were switched to a vitamin D-replete diet at 8 weeks of age (Fig. 1). Experiments were performed over a 12-month period between November 2011 and November 2012.

### Measurement of serum and BALF levels of 25-hydroxyvitamin D (25(OH)D)

Serum and BALF 25(OH)D levels were measured using IDS EIA ELISA kits (Immunodiagnostic Systems Ltd, Fountain Hills, AZ) as described by the manufacturer (limit of detection was 5–7 nmol/L).

### Determining bacterial loads in lung samples

Following the BALF procedure (see below), the left lobe of each lung was minced using a sterile scalpel blade. A 2 mm^3^ sample of the lung was snap-frozen in liquid nitrogen. DNA was extracted from lung samples using the DNeasy Blood and Tissue DNA extraction kit as described by the manufacturer (Qiagen). Universal 16S rRNA primers (F primer = 5′-TCCTACGGGAGGCAGCAG T-3′; R primer = 5′-GGACTACCAGGGTATCTAATCCTGTT-3′ [26]) were used to detect bacteria in DNA samples using the Power SYBR Green PCR Master Mix as described by the manufacturer (Applied Biosystems, Calsbad, USA). These ‘universal primers’ have broad specificity to detect conserved regions of 16S rDNA from 34 bacterial species encompassing most bacterial groups [26]. An 18S rRNA endogenous control including a FAM-MGB probe was used as the internal control for this assay using conditions described by the manufacturer (Applied Biosystems).

### Characterising the bacterial lung microbiome

To profile the microbial community of the lungs, we amplified the variable region V3-V4 of the 16S rRNA gene as previously reported [17], with modifications, using the primer pair of 341 F and 806R. The PCR mix was composed of 5 μl 5x Phusion buffer HF (7.5 mM MgCl_2_, Finnzymes, Finland), 0.5 μl 10 mM dNTPs, 1.25 μl 10 μM of each primer, 0.25 μl DNA polymerase (Hotstart Phusion 540 L, 1 unit/μl Finnzymes) and 3.5 μl template. The PCR program started with 98 °C (2 min), followed by 35 cycles of denaturation at 98 °C (5 s), annealing at 55 °C (30 s) and strand elongation at 60 °C (60 s). The PCR was finalised by a single elongation step at 72 °C for 5 min and than cooled to 4 °C. The size of the PCR product was evaluated using gel electrophoresis. The fragment was then excised and purified using the Montage Gel Extraction Kit (Merck Millipore) with negative controls also excised at the position of the expected fragment size. Adaptors were added to the amplicons in a second PCR run under the same conditions as PCR I with a reduced cycle number of 15. The sequences were generated with GS FLX Titanium (454 Life Sciences, Roche). Two sequencing runs were performed, with the machine cleaned between runs (using standard protocols) and the second run then proceeding, with each run containing samples from the ‘naïve’ and ‘OVA-induced allergic airway disease’ datasets. The 1,475,452 reads were trimmed for low quality (minimal quality score = 25) using the Qiime pipeline version 1.5.0 [27]. Only sequences with a minimal length of 200 bp were considered, the sequences were denoised using the Ampliconnoise algorithm [28]. Chimeras were removed using the Uchime algorithm [29]. Operational taxonomic units (OTUs) were picked *de novo* from quality-checked reads and clustered at 97 % sequence similarity using Uclust. Taxonomy was assigned using the RDP classifier (version 2.2) method and Greengenes as reference database [30]. After data treatment, 280,699 reads from the ‘naïve dataset’, and 330,733 reads from the ‘OVA-allergic airway disease’ dataset, were used for down stream analysis. To adjust for differences in sequencing depth between samples the OTUs were normalised using the cumulative sum-scaling method available in the metagenomeSeq Bioconductor package [31].

### Bronchoalveolar lavage fluid for assessment of ß-defensin-2 (mBD2)

A total of 1 ml BALF was collected for each mouse as described previously [32]. Levels of mBD2 in BALF were detected using an ELISA kit and method supplied by USCN Life Science Inc (Wuhan, China).

### Sensitisation and challenge of mice with ovalbumin

Ovalbumin (OVA) (Sigma Chemical Company, St Louis, MO, USA) was mixed with an aluminium hydroxide suspension (Alum, Serva, Heidelberg, Germany). This OVA/Alum solution was diluted in 0.9 % saline to sensitise and boost mice intraperitoneally (200 μl) with 1 μg OVA and 0.2 mg Alum as previously described, with mice sensitised at 12 weeks of age and boosted at 14 days of age [24]. For respiratory challenge, mice were placed in a Perspex box and inhaled a 1 % OVA-in-saline (1 mg/ml) aerosol delivered using an ultrasonic nebuliser (UltraNebs, DeVilbiss, Somerset, PA, USA) for 30 min. This was performed once, 7 days after the OVA/Alum boost. Lungs and BALF was obtained 24 h after the aerosol challenge.

### Bronchoalveolar lavage fluid for assessment of inflammatory cells

BALF cells (5 × 10^5^) were spun onto glass slides using a cytocentrifuge and differential counts of inflammatory cells performed after staining cells with the DIFF-Quik Stain Set 64851 (Lab Aids, Narrabeen, NSW, Australia) as per the manufacturer’s instructions. At least 300 cells were counted for each sample from ≥3 independent fields of view (x100).

### Measurement of circulating immune cells

Blood was obtained from mice and immediately added to K_2_EDTA-coated Microtainer tubes (BD, Franklin Lakes, NJ) to prevent clotting. The proportions of circulating neutrophils and monocytes within the white blood cell population was determined using the ADVIA® 120 Haematology System (Siemens Healthcare Diagnostics Inc, Tarrytown, NY).

### Statistical analyses

Data were compared using one-way ANOVA or an unpaired two-way student’s *t* test or correlated (Spearman’s rank correlation) using the Prism 5 for Mac OS X statistical analysis program. Differences were considered significant with a *p-*value <0.05. We used the non-parametric Kruskal-Wallis test (*p* < 0.05) to test for significant differences in OTU abundance. Treatment effects on the complex microbial communities in the lung were observed by applying the vegdist function (part of the R vegan package) on the raw metagenomeSeq normalised OTU abundance table to generate the Bray-Curtis dissimilarity between samples.

The lungs of specific-pathogen-free mice contain little microbial DNA, and as recently described for microbiome studies [33] are of high risk of contamination by bacterial DNA present in molecular biology reagents used for high throughput sequencing. These can especially contribute towards incorrect interpretations of cluster analyses. However, simply removing OTUs from the abundance table might introduce bias by potentially removing ‘real’ OTUs present in the sample (e.g. *Escherichia* spp.). Therefore, the entire cluster analysis that was based on the metagenomeSeq normalised OTU table was performed twice; first with, and then without 126 OTUs detected in the blank reagents and listed in the OTU table that contained afterwards 5,635 OTUs for downstream analysis. Most (96 %) of the contaminating OTUs were taxonomically assigned to the halophilic marine genus *Halomonas*. The rest were *Shewanella*, *Delftia* and *Stenotrophomonas.* Unless stated differently we used the cleaned microbial OTU table describing the lung microbiome according to [34].

The Bray-Curtis dissimilarity test takes microbial diversity and relative abundance into account. Grouping of samples was visualised by using ordination applying non-metric multidimensional scaling (NMDS) generated in the R vegan package [35]. Microbial clustering was further evaluated with the analysis of similarity (ANOSIM), which takes the ranked dissimilarities (Bray-Curtis) of samples belonging to the same treatment group and compares it to the distance of samples of a different treatment [36]. The closer the ANOSIM generated *R*-value was to 1, the larger the variation between microbial treatments, whereas 0 indicates no difference. The results were than tested for significance by 999 permutations with a 5 % significance level. Individual variation of selected microbes was displayed with the Euclidean distance in a one-sided dendrogram (Heatmap) based on the log-transformed metagenomeSeq normalised OTU counts. The percentage of each OTU was relative to the number of OTUs for each sample.

## Results

### Dietary vitamin D increased circulating but not BALF 25(OH)D levels

**Fig. 2:**
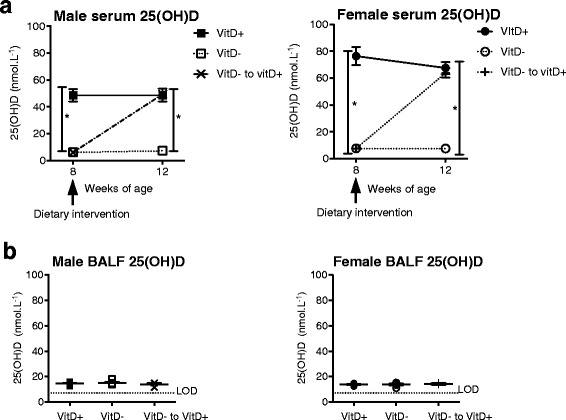
Vitamin D deficiency reduced serum 25(OH)D levels but had no effect on BALF 25(OH)D levels. Female BALB/c mice (dams) were fed vitamin D-containing (VitD+) or vitamin D-null (VitD-) diets from 3 weeks of age and used to produce offspring. A subgroup of the vitamin D-deficient offspring was fed a vitamin D-supplemented diet from 8 weeks of age (VitD−to vitD+). In (**a**), serum 25(OH)D levels in offspring at 8 and 12 weeks of age (mean ± SEM for ≥5 mice per group). In (**b**) BALF 25(OH)D levels in offspring at 12 weeks of age (mean ± SEM for ≥3 mice per group, the broken line indicates the level of detection (LOD) for 25(OH)D; 7 pg/ml). (*p < 0.05)

Circulating serum levels of 25(OH)D were measured in 8-week old offspring born to vitamin D-replete and -deficient dams (Fig. 2a). Serum 25(OH)D levels were <20 or ≥50 nmol.L^−1^ in offspring fed a vitamin D-deficient or -replete diet, respectively. When vitamin D-deficient offspring were fed a vitamin D-containing diet for four weeks, their serum 25(OH)D increased to levels equivalent to mice fed a vitamin D-containing diet throughout the experiment (Fig. 2a). BALF 25(OH)D levels were low, although greater than the detection limit of the ELISA and not affected by dietary vitamin D (Fig. 2b).

### The effects of dietary vitamin D on bacterial loads in the lungs of naive mice

**Fig. 3:**
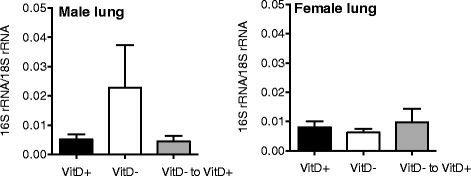
The effects of vitamin D deficiency on lung bacterial load in naïve mice. Female BALB/c mice (dams) were fed vitamin D-containing (VitD+) or vitamin D-null (VitD−) diets from 3 weeks of age and used to produce offspring. A subgroup of the vitamin D-deficient offspring was fed a vitamin D-supplemented diet from 8 weeks of age (VitD−to VitD+). Bacterial DNA levels were determined in the lungs of naïve mice using a PCR with universal primers for detection of bacterial 16S rRNA gene (mean + SEM for 10–15 mice/treatment)

There was a trend similar to our previously published findings (24) for increased bacterial load in the lungs of vitamin D-deficient male mice, although this was not statistically significant (One-way ANOVA, F = 1.481, *p* = 0.245; Fig. 3). Bacterial load was quantified using a PCR with 16S rRNA primers capable of amplifying DNA of most bacterial species.

### Dietary vitamin D had a sex-specific effect on the lung microbiome of naive mice

**Fig. 4:**
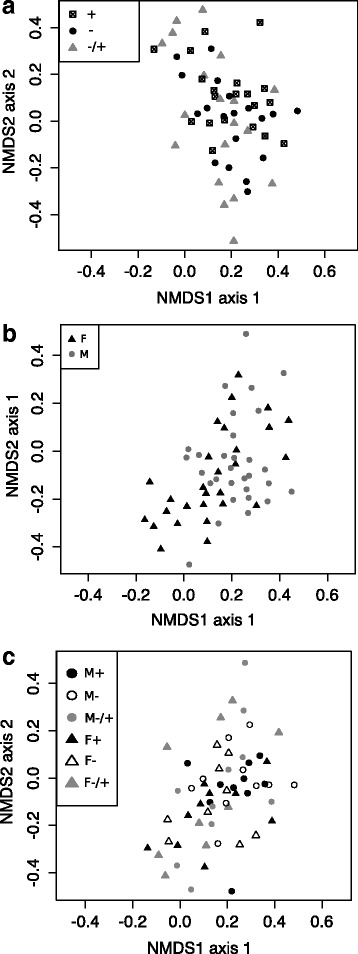
The lung microbiomes of vitamin D-replete and -deficient naïve mice were similar. Female BALB/c mice (dams) were fed vitamin D-containing (+) or vitamin D-null (−) diets from 3 weeks of age and used to produce offspring. A subgroup of the vitamin D-deficient offspring was fed a vitamin D-supplemented diet from 8 weeks of age (−/+). Non-metric multidimensional scaling (NMDS) plots depict the dissimilarity of the detected operational taxonomic units (OTUs) from the lungs, with results shown for clustering by (**a**) diet, (**b**) sex, or (**c**) all (M = male, F = female; *n* = 9-10/treatment)

**Fig. 5:**
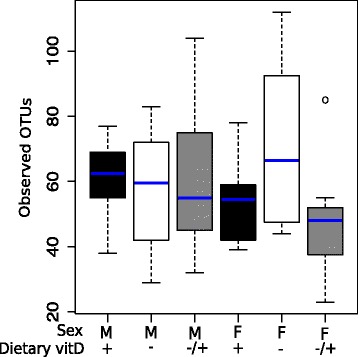
Dietary vitamin D did not affect the diversity of the lung microbiome. Female BALB/c mice (dams) were fed vitamin D-containing (+) or vitamin D-null (−) diets from 3 weeks of age and used to produce male (M) and female (F) offspring. A subgroup of the vitamin D-deficient offspring was fed a vitamin D-supplemented diet from 8 weeks of age (−/+). Shown are the number of operational taxonomic units (OTUs) observed at a sequencing depth of 1,200 per animal, with no significant effect of sex or vitamin D. Data is shown with the median as a blue line, the first or third quartiles as the upper and lower boxes (respectively) and the upper and lower whiskers show the 1.5 times of the interquartile distance and minimum and maximum data (*n* = 9–10/treatment, with an outlier denoted as a circle)

**Fig. 6:**
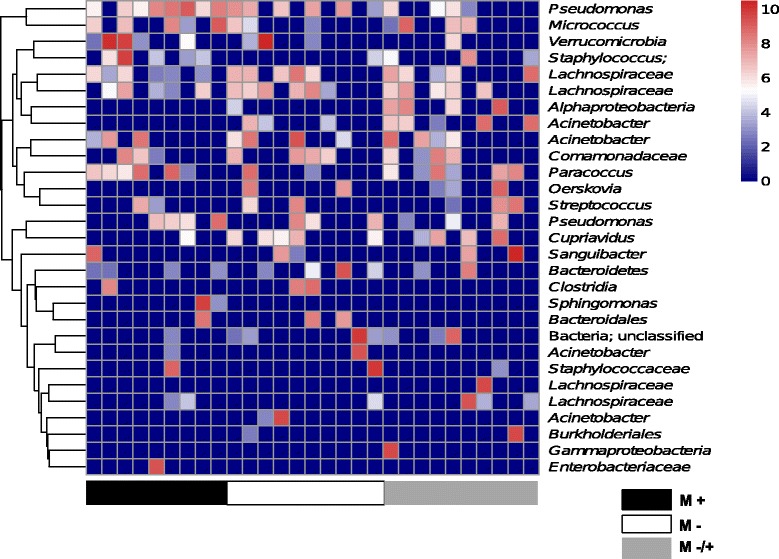
The abundance of bacterial operational taxonomic units was minimally affected by vitamin D deficiency. Female BALB/c mice (dams) were fed vitamin D-containing (+) or vitamin D-null (−) diets from 3 weeks of age and used to produce offspring. A subgroup of the vitamin D-deficient offspring was fed a vitamin D-supplemented diet from 8 weeks of age (−/+). Shown is a heat map of the of operational taxonomic units (OTUs) significantly detected in the lungs of male (M) naïve offspring. OTU counts were log-transformed; with red denoting increased frequency of a given OTU, and dark blue no occurrence (*n* = 9-10/treatment)

We used high-throughput sequencing to determine how vitamin D might affect the composition of bacteria in the lung microbiome. After data treatment (see methods) there was an average read distribution of 4,803 sequences per naïve animal. We first evaluated if dietary vitamin D had a significant impact on the microbial composition enumerating the dissimilarity of the OTUs. As shown in NMDS plots, there was no clustering by diet (Fig. 4a), sex (Fig. 4b), or the combination of both (Fig. 4c). We then evaluated the relative abundance of OTUs with 23–112 OTUs identified/mouse (Fig. 5). Neither dietary vitamin D nor sex significantly affected bacterial diversity in the lungs as defined by the relative number (Fig. 5, Kruskal-Wallis Test, *p* > 0.05) of OTUs. In Fig. 6, a heat map was used to show the most frequent OTUs identified from the lungs of male mice. There was very little effect of dietary vitamin D on the relative proportion of each OTU (Fig. 6), with similar findings in female mice (data not shown), and little difference in bacterial variation between treatments identified using ANOSIM analysis, except in mice fed a vitamin D-containing diet throughout the experiment (Table 1).

**Table 1:** Comparison of the lung microbiome within groups with analysis of similarity (ANOSIM) examining the effects of sex or dietary vitamin D in naïve mice

Factor	Groups compared	ANOSIM R	*p*-value
Sex	F+ vs M+	0.151	0.017*
	F- vs M−	−0.065	0.822
	F−/+ vs M−/+	−0.033	0.659
Vitamin D	M+ vs M−	−0.044	0.811
	M+ vs M−/+	0.049	0.151
	M−/+ vs M−	0.008	0.379
	F+ vs F−	−0.051	0.681
	F+ vs F−/+	0.013	0.406
	F−/+ vs F−	−0.047	0.738

*Significant clustering, *p* < 0.05

F = Female

M = Male

(+) = fed vitamin D-supplemented diet throughout life

(−) = fed vitamin D-null diet throughout life

(−/+) = fed vitamin D-null diet until 8 weeks of age and then vitamin D-supplemented diet

**Fig. 7:**
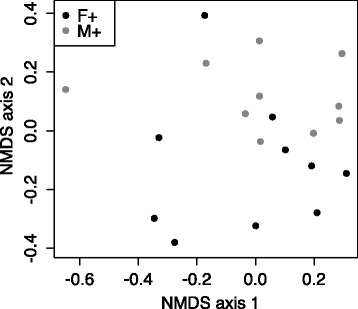
The lung microbiomes of vitamin D-replete and -deficient naïve mice were similar. Female BALB/c mice (dams) were fed vitamin D-containing (+) or vitamin D-null (−) diets from 3 weeks of age and used to produce offspring. A subgroup of the vitamin D-deficient offspring was fed a vitamin D-supplemented diet from 8 weeks of age (−/+). Non-metric multidimensional scaling (NMDS) plots depict the dissimilarity of the detected operational taxonomic units (OTUs) from the lungs, with results shown for clustering for mice fed the vitamin D+ diet only. (M = male, F = female; *n* = 9–10/treatment)

We observed a significant separation (NMDS cluster) between female and male mice that were fed the vitamin D-supplemented diet throughout the experiment (Fig. 7). This difference was attributed to increased representation of an *Acinetobacter* OTU in female mice, which varied significantly between the mice (Wilcoxon Rank Sum test, *p* = 0.02), together with a single low-abundant OTU that could only be annotated as bacteria. Reduced carriage of the *Acinetobacter* OTU was observed in male mice (2/10 mice; ≤5 % of sequences in the observed sequences; mean = 0.7 %), compared to female mice (7/10 mice; ≤40 % of sequences in the observed sequences; mean = 7.0 %) fed a vitamin D-containing diet throughout the experiment.

### Antimicrobial levels in the lungs were enhanced by dietary vitamin D in both male and female mice

**Fig. 8:**
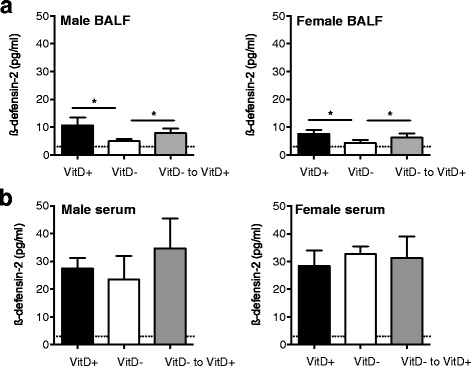
Vitamin D deficiency reduced ß-defensin-2 (mBD2) protein levels in the BALF of naïve mice. Female BALB/c mice (dams) were fed vitamin D-containing (VitD+) or vitamin D-null (VitD-) diets from 3 weeks of age and used to produce offspring. A subgroup of the vitamin D-deficient offspring was fed a vitamin D-supplemented diet from 8 weeks of age (VitD− to VitD+). ß-defensin-2 protein levels were determined in the BALF (**a**) (*n* = 10 mice/treatment) and serum (**b**) (n ≥ 3 mice/treatment) of naïve mice (mean + SEM) (*p < 0.05, the broken line indicates the level of detection (LOD) for ß-defensin-2; 3 pg/ml)

In both male and female mice, dietary vitamin D increased ß-defensin-2 (mBD2) protein levels in BALF, with the effects of early-life vitamin D-deficiency reversed by dietary vitamin D (Fig. 8a). There was no effect of dietary vitamin D on serum mBD2 levels (Fig. 8b). Levels of mBD2 were significantly greater in serum than BALF. These results suggest that the sex-dependent effects of vitamin D on *Acinetobacter* in the lungs were not due to differences in the expression of mBD2 as dietary vitamin D increased levels to a similar degree in the BALF of male and female mice.

### Serum 25(OH)D levels inversely correlated with the most prominent OTU of Pseudomonas in the lung

**Fig. 9:**
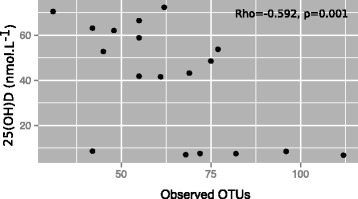
Number of operational taxonomic units in lungs inversely correlated with serum 25(OH)D. Female BALB/c mice (dams) were fed vitamin D-containing (+) or vitamin D-null (−) diets from 3 weeks of age and used to produce offspring. A subgroup of the vitamin D-deficient male and female offspring was fed a vitamin D-supplemented diet for 4 weeks from 8 weeks of age (−/+). A significant inverse correlation was observed between the number of operational taxonomic units (OTUs) in the lungs and circulating 25(OH)D levels (for *n* = 18 mice for whom both measures were obtained)

In a separate analysis, we identified an inverse correlation between serum 25(OH)D levels and the total number of OTUs (Fig. 9, Spearman’s rho = −0.592, *p* = 0.001). When each sex was considered separately, some evidence for an inverse correlation was observed between total OTUs in the lungs and serum 25(OH)D levels in females (Spearman’s rho = −0.642, *p* = 0.052) but not males (Spearman’s rho = 0.463, *p* = 0.238). When examining individual OTUs in the lungs, there was a strong negative correlation between 25(OH)D levels in serum and an unspecified *Pseudomonas* OTU (Spearman’s rho = −0.556, *p* = 0.016), with a marginal evidence observed in females (Spearman’s rho = −0.611, *p* = 0.081) but not males (Spearman’s rho = −0.343, *p* = 0.366). Some evidence for a negative correlation was observed between serum 25(OH)D and all *Pseudomonas* OTUs (Spearman’s rho = −0.429, *p* = 0.07). However, there was no evidence of a relationship between total OTUs or the unspecified *Pseudomonas* OTUs in the lung and mBD2 levels in BALF (total OTUs; Spearman’s rho = 0.109, *p* = 0.429; *Pseudomona*s OTU Spearman’s rho = 0.202, *p* = 0.138 for BALF) or serum (total OTUs; Spearman’s rho = 0.114, *p* = 0.673, *Pseudomona*s OTU Spearman’s rho = 0.114, *p* = 0.673).

### The lung microbiomes of naïve mice, and, ovalbumin (OVA)-sensitised and -challenged mice clustered separately

**Fig. 10:**
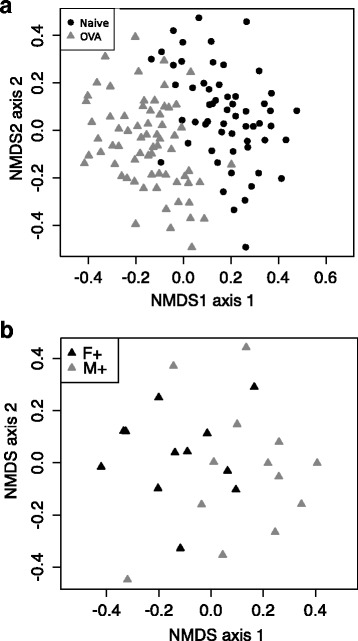
Ovalbumin-induced allergic airway disease substantially modified the lung microbiome. The lung microbiomes of naïve mice (naïve, *n* = 56) were compared with those with ovalbumin (OVA)-induced allergic airway disease (OVA, *n* = 68), 24 h after respiratory challenge with OVA, with results combined for all naïve or all OVA mice. To induce allergic airway disease, mice were injected with OVA and Alum at 12 weeks of age, boosted with OVA and Alum at 14 weeks of age, and then administered a respiratory challenge with OVA at 15 weeks of age. Lungs were obtained 24 h later. In (**a**), a non-metric multidimensional scaling (NMDS) plot depicts the dissimilarity of the detected lung of operational taxonomic units (OTUs) for all mice, and in (**b**) for mice only fed the vitamin D-supplemented diet (+). (M = male, F = female; *n* = 9–10/treatment)

We hypothesised that allergic sensitisation and challenge with OVA (to induce allergic airway disease) would significantly change the microbiota composition of the lungs. The relative effects of the induction of OVA-induced allergic airway versus that of vitamin D deficiency (alone) on the lung microbiome are unknown. To better understand the magnitude of the observed effect of dietary vitamin D on the lung microbiome, we compared its capacity to regulate the lung microbiome to the effects of the induction of allergic airway disease. Mice were intraperitoneally sensitised and boosted with ovalbumin (OVA) (1 μg) and Alum hydroxide suspension (Alum, adjuvant, 0.2 mg) at 12 and 14 weeks of age (respectively), which was followed by a respiratory challenge at 15 weeks of age with aerosolised OVA-in-saline (1 mg/ml) [24]. Lungs were sampled 24 h after the challenge. We compared the overall microbial community from the lungs of the naïve mice characterised above (*n* = 58; 4,803 sequences per naïve mouse) with the lungs of mice with OVA-induced allergic airway disease (*n* = 68; 4,469 sequences per mouse). As shown in Fig. 10a, the lung samples of naïve mice and mice with allergic airway disease clustered significantly apart, which was confirmed using a compositional analysis to compare the difference in numbers of observed bacterial OTUs (Bray-Curtis, R = 0.334, *p* = 0.001). A NMDS analysis confirmed that observed differences were not induced by performing two sequencing runs (data not shown). Bacterial diversity was not affected with the mean number of OTUs for naïve mice or mice with allergic airway disease (naïve, 138 + 5.1; allergic airway disease, 128 + 5.4; mean + SEM, *p* = 0.132), respectively.

### The lung microbiome was significantly altered by allergic airway disease

**Fig. 11:**
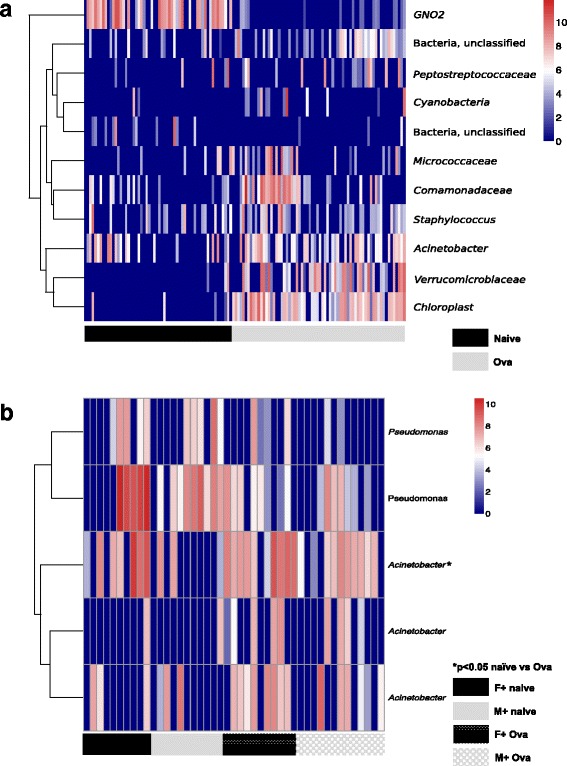
Ovalbumin-induced allergic airway disease substantially modified the lung microbiome. The lung microbiomes of naïve mice (naïve, *n* = 56) were compared with those with ovalbumin (OVA)-induced allergic airway disease (OVA, *n* = 68), 24 h after respiratory challenge with OVA, with results combined for all naïve or all OVA mice. To induce allergic airway disease, mice were injected with OVA and Alum at 12 weeks of age, boosted with OVA and Alum at 14 weeks of age, and then administered a respiratory challenge with OVA at 15 weeks of age. Lungs were obtained 24 h later. In (**a**), a heat map shows OTUs significantly detected in the lungs of naïve and OVA-sensitised and -challenged mice. OTU counts were log-transformed; with red denoting increased frequency of a given OTU, and dark blue no occurrence. In (**b**), a heat map compares the sex-specific differences in detected *Pseudomonas* and *Acinetobacter* OTUs in naïve mice and mice with OVA-induced allergic airway disease (M = male, F = female), with a significant difference (*p < 0.05) denoting significant more of the most abundant *Acinetobacter* OTU in male mice with OVA-induced allergic airway disease (for mice fed a vitamin D-containing diet throughout the experiment)

The strength of the difference between the lung microbiomes of naïve mice, and, mice with allergic airway disease was quantified by ANOSIM. A significant difference was identified (Table 2). As for naïve mice (Fig. 7), a significant separation (NMDS cluster) was observed between female and male mice that were fed the vitamin D-supplemented diet throughout the experiment with OVA-induced allergic airway disease (Fig. 10b; Table 3 for ANOSIM bacterial variance). There was a significant effect of allergic airway disease on the relative proportion of OTUs detected in mice. The most abundant OTUs detected, which each accounted for >1 % of the sequences after normalization, are shown for the naïve mice, or, mice with allergic airway disease in Fig. 11a. Proportions of the environmental microbial group GNO2 (Naïve, mean = 9.5 %; OVA, mean = 0.0 %) and an unassigned bacterial OTU (Naïve, mean = 1.1 %; OVA, mean = 0.0 %) were reduced in mice with OVA-induced allergic airway disease, while *Acinetobacter* (Naïve, mean = 1.29 %; OVA, mean = 1.8 %), *Chloroplast* (Naïve, mean = 0.1 %, OVA, mean = 5.0), *Comamonadaceae* (Naïve, mean = 0.1 %; OVA, mean = 4.0.%), *Cyanobacteria* (Naive, mean = 0.1 %; OVA, mean = 1.0 %) *Staphylococcus* (Naïve, mean = 0.5 %; OVA, mean = 1.2 %), *Micrococcaceae* (Naïve, mean = 0.1 %; OVA, mean = 1.8 %) and *Verrucomicrobiaceae* (Naïve, mean = 0.1 %; OVA, mean = 7.6 %) OTUs, and an OTU assigned to *Peptostreptococcaceae* (Naïve, mean = 1.0 %; OVA, mean = 1.5 %) were increased by the induction of allergic airway disease. The *Acinetobacter* OTU significantly increased in the OVA samples was not the same *Acinetobacter* OTU identified as responsible for largely driving the difference between male and female mice fed a vitamin D-containing diet throughout the experiment. There was no significant difference in the number of any *Pseudomonas* OTUs detected in the lungs of naïve mice, or, mice with allergic airway disease. Together, these results suggest that the effects of allergic airway disease on the lung microbiome of mice far exceeded those of vitamin D deficiency.

**Table 2:** Group comparison of the lung microbiomes of naïve mice, with those from mice with OVA-induced allergic airway disease within treatments for analysis of similarity (ANOSIM)

Factor	Groups	ANOSIM R	*p*-value
OVA-induced allergic airway disease	M+	0.434	0.001*
	M−	0.394	0.001*
	M−/+	0.354	0.001*
	F+	0.243	0.002*
	F−	0.281	0.001*
	F−/+	0.31	0.001*

*Significant clustering, *p* < 0.05

F = Female

M = Male

(+) = fed vitamin D-supplemented diet throughout life

(−) = fed vitamin D-null diet throughout life

(−/+) = fed vitamin D-null diet until 8 weeks of age and then vitamin D-supplemented diet

**Table 3:** Comparison of the lung microbiomes of mice with OVA-induced allergic airway disease with analysis of similarity (ANOSIM) examining the effects of sex or dietary vitamin D

Factor	Groups compared	ANOSIM R	*p*-value
Sex	F+ vs M+	0.149	0.009*
	F− vs M−	0.067	0.137
	F−/+ vs M−/+	−0.001	0.481
Vitamin D	M+ vs M−	0.008	0.418
	M+ vs M−/+	0.028	0.228
	M−/+ vs M−	0.076	0.115
	F+ vs F−	0.057	0.141
	F+ vs F−/+	0.039	0.219
	F−/+ vs F−	0.055	0.178

*Significant clustering, *p* < 0.05

F = Female

M = Male

(+) = fed vitamin D-supplemented diet throughout life

(−) = fed vitamin D-null diet throughout life

(−/+) = fed vitamin D-null diet until 8 weeks of age and then vitamin D-supplemented diet

Even though OVA-induced allergic airway disease substantially altered the lung microbiome, the sex-specific effect on the abundance of *Acinetobacter* OTUs was still apparent in mice fed exclusively a vitamin D-containing diet (Table 3). *Acinetobacter* OTUs were more frequent in OVA-induced allergic airway disease than naïve mice (Fig. 11b). There were a further 11 OTUs varied in their frequency when the lung microbiomes of female and male mice with OVA-induced allergic airway disease were compared. However, these were present at a very low frequency (<0.01 % of the total normalised sequences). There was no effect of OVA-induced allergic airway disease on the proportions of the dominant *Acinetobacter* OTU in female mice; however, induction of OVA-induced allergic airway disease increased the proportions of this OTU in male mice. For example, for male mice fed the vitamin D-containing diet throughout the experiment, the percentage of this OTU increased from 0.69 + 0.51 (mean + SEM) in naïve mice to 4.7 + 1.5 in mice with OVA-induced allergic airway disease (*p < 0.05, *n* = 10–13 mice/treatment). There was no significant correlation between the number of *Acinetobacter* OTUs and 25(OH)D or mBD2 levels in BALF or serum (Spearman *p* > 0.05, data not shown).

### OVA-induced allergic airway disease had a substantially greater effect on inflammatory cells in BALF than vitamin D deficiency

**Fig. 12:**
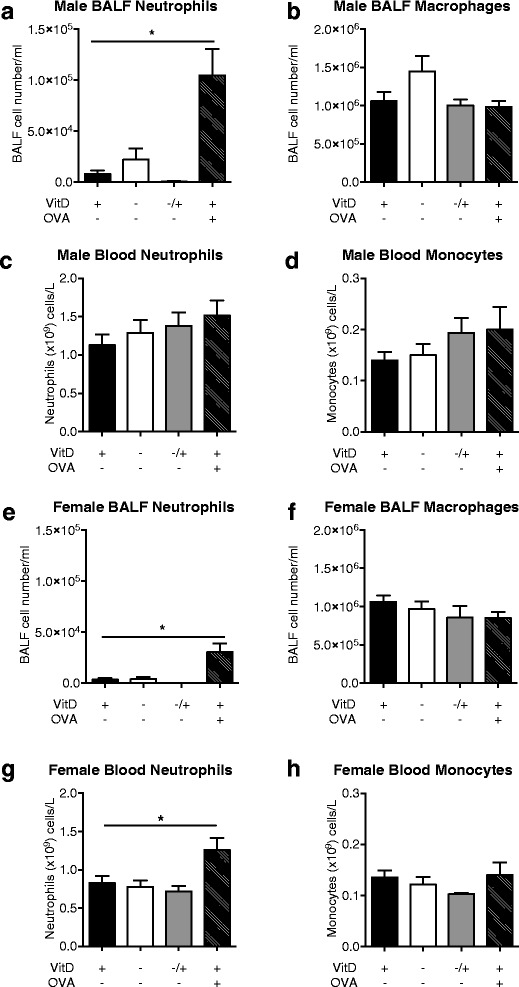
Allergic airway disease had a substantially greater effect on bronchoalveolar lavage neutrophil numbers than vitamin D deficiency. Neutrophil and macrophage numbers in the bronchoalveolar lavage (BALF) and blood of naïve mice fed a vitamin D (VitD)-replete (+) or -deficient (−) or initially vitamin D-deficient then replete diet (−/+) diet were compared with those of vitamin D-replete mice with OVA-induced allergic airway disease, 24 h after respiratory challenge with OVA. For OVA-sensitisation, mice were injected with OVA and Alum at 12 weeks of age, boosted with OVA and Alum at 14 weeks of age, and then administered a respiratory challenge with OVA at 15 weeks of age. BALF or blood was obtained 24 h later. In (**a**-**d**) results from males, and (**e**-**h**) results from female mice. In (**a**) and (**e**) neutrophils, and (**b**) and (**f**) macrophages in BALF are shown (n ≥ 14 mice/treatment). In (**c**) and (**g**) neutrophils, and (**d**) and (**h**) monocytes per ml of blood are depicted (*n* = 5–10 mice/treatment). Data is shown as mean + SEM (*p < 0.05)

The effects of vitamin D deficiency and the induction of OVA-induced allergic airway disease on numbers of neutrophils and macrophages/monocytes in the BALF or blood were then compared. Substantially increased BALF neutrophil numbers were observed in both male (Fig. 12a) and female (Fig. 12e) mice with OVA-induced allergic airway disease as expected; however, there was only a marginal effect of vitamin D deficiency. There was also a significant increase in neutrophil numbers in the blood of female mice with OVA-induced allergic airway disease, with no effect of vitamin D deficiency (Fig. 12f). Neither treatment significantly modulated macrophage levels in BALF (Fig. 12b, f), nor monocytes levels in blood (Fig. 12d, h). These data suggest that there is an important interplay between respiratory inflammation and the lung microbiome.

## Discussion

We observed modest effects of dietary vitamin D on the bacterial composition of the lung microbiome. Serum 25(OH)D levels inversely correlated with the number of OTUs detected, and more specifically the presence of an unidentified *Pseudomonas* OTU in the lungs of naïve mice. These results suggest that vitamin D sufficiency limited the number of respiratory pathobionts (like *Pseudomonas*) rather than increasing the number of protective commensals in the lungs. We also observed a sex difference in mice fed the vitamin D-containing diet throughout the experiment, with differences observed largely limited to a single *Acintobacter* OTU. The induction of allergic airway disease altered the lung microbiome composition to a degree that far exceeded any of the more subtle effects of vitamin D deficiency. More external environmental microbes, such as Cyanobacteria and Chloroplast, were detected in mice with allergic airways disease as well as OTUs previously identified in the lung microbiome, such as *Staphylococcus*, *Peptostreptococcaceae* and *Acinetobacter*. BALF neutrophil numbers were increased in mice of both sexes by ~10-fold with the induction of OVA-induced allergic airway disease, while the effects of vitamin D deficiency on these cell numbers in BALF were modest.

There were sex-specific differences in the lung microbiomes of mice fed a vitamin D-containing diet throughout the experiment. The *Acinetobacter* OTU, which accounted these effects, was distinct from the *Acinetobacter* OTU modulated by OVA-induced allergic airway disease. The nature of the sex-specific effect of vitamin D on the *Acinetobacter* OTUs differed, depending on whether mice were naïve or had OVA-induced allergic airway disease. We have previously shown sex-specific clustering of the lung microbiome in a different mouse strain (C57Bl/6) using denaturing gradient gel electrophoresis [37]. *Acinetobacter* may be a common commensal of human [38] and mouse lungs [17]. This genus may colonise the lungs early in life and has been linked to protection from allergy [39–41]. The switching of the direction of the sex-specific effects may reflect differences in the severity of OVA-induced allergic airway disease in male and female mice that are regulated by vitamin D, as previously observed [24]. The sex-specific effects on the lung microbiome were not reproduced by supplementing initially deficient mice with vitamin D, supporting the hypothesis that colonization with *Acinetobacter* species in the lungs occurs early in life.

As we have previously observed, 25(OH)D levels were reduced in male mice as compared to female mice fed a vitamin D-containing diet (see also [24, 25, 42, 43]). This observation is explained by increased renal expression of 24-hydroxylase (CYP24A1), the enzyme responsible for breaking down active 1,25(OH)_2_D, in male mice [42]. Hormones influence other effects of vitamin D, with reports of functional synergies between 1,25(OH)_2_D and 17-β-estradiol in T cells [44, 45], and differential regulation of 1,25(OH)_2_D-modulated pathways by testosterone in males and females [45, 46]. Testosterone increased Foxp3 (regulatory gene) expression in T cells from women, but not men [46] and 1,25(OH)_2_D more potently induced CD4 + CD25 + Foxp3+ (regulatory) T cells from PBMCs of female than male subjects [45], although we observed similar reductions in T_Reg_ cell percentages in the skin-draining lymph nodes of vitamin D-deficient male and female mice [47]. These effects of estrogen and testosterone on immune cell function suggest that females may be more resilient to the effects of vitamin D deficiency than males.

The inverse relationship between lung *Pseudomonas* and serum 25(OH)D could be related to increased levels of mBD2 in BALF of vitamin D-sufficient mice. mBD2 is a member of the defensin family of peptides, which have well-established killing effects on Gram-negative bacteria like *Pseudomonas*. mBD2 exhibits significant protein sequence homology with human ß-defensins 1 and 2 [48] and is expressed by a variety of epithelial cells (including tracheal) as well some immune cells (reviewed in [49]). mBD2 expression is induced during infection with Gram-negative bacteria, their products (e.g. lipopolysaccharide), and various proinflammatory cytokines (e.g. tumour necrosis factor) [48, 50]. Lipopolysaccharide may also drive the production of active 1,25(OH)_2_D from circulating 25(OH)D, resulting in synthesis of ß-defensins [51]. mBD2 is an immunoregulatory molecule, and its expression improves bacterial resistance by promoting proinflammatory cytokine expression through activation of toll-like receptor-4 [50]. Using immunohistochemistry, other researchers have detected only minimal concentrations of mBD2 in the lungs of BALB/c mice with OVA-induced allergic airway disease [52]. In addition, reduced levels of cathelin-related antimicrobial peptide (the mouse equivalent of cathelicidin) were detected in mice with OVA-induced allergic airway disease in comparison to non-sensitised mice [53]. These findings suggest that OVA-induced allergic airway disease may reduce the expression of antimicrobials like mBD2 levels in the lungs.

Regulation of mBD2 by vitamin D may significantly inhibit specific bacteria like *Pseudomonas*. Wu et al. demonstrated that mBD2 was required for host resistance against corneal infection with *Pseudomonas aeruginosa* [49]. These bacteria were more frequently detected in the sputum of vitamin D-deficient patients with bronchiestasis than -replete patients [54]. However, in another study there was no difference in the incidence of *P. aeruginosa* infection in children with cystic fibrosis that were vitamin D-deficient or -sufficient (25(OH)D > 30 μg/L) [55]. Active 1,25(OH)_2_D (10 nM) induced antimicrobial activity in bronchial epithelial cells against *P. aeruginosa* [56]. While we observed a significant inverse relationship between total OTUs or numbers of an unspecified *Pseudomonas* OTU and serum 25(OH)D, there was no inverse association with mBD2 in BALF. It is likely that another explanation, such as compromised epithelial integrity [6, 8], is responsible for the observed inverse relationship between serum 25(OH)D and lung OTUs.

In addition to *Pseudomonas*, we identified microbes in the lungs of our naïve mice that were previously detected in the lungs of humans [57] and other mice [17] including OTUs of *Micrococcus*, *Staphylococcus*, *Cupriavidus* and *Streptococcus*. The microbiome dataset from the lung tissue of naive mice (in particular) was very sparse, with few OTUs found among all lung samples (~130–140 OTUs/mouse). We obtained lung samples directly from the thoracic cavity of euthanised specific-pathogen-free mice, limiting possible contamination from the upper respiratory tract and oral cavity. It is possible that the modest effects of dietary vitamin D were due to the scarcity of the collected DNA. However, we believe this is not likely, due to the rigorous use of negative controls to identify OTUs of contaminating bacteria [33], our observations of association between circulating 25(OH)D levels and lung OTUs, and the significant microbial shift induced by the induction of allergic airway disease.

Methodological considerations are important, as we have previously shown differences in the microbial diversity of BALF and lung tissue [17]. With the small biomass of bacteria expected in the lungs of specific-pathogen-free naïve mice [58], there could also be problems associated with low yield DNA introduced through bacterial contamination of commercially purchased products (eg. in molecular-grade water or PCR reagents, [34]). As described by Salter et al. [34], reagents and extractions kits used for 16S amplicon library preparation can mask real biological effects and or increase false results. This is particularly a problem with lung samples as the bacterial yield is low, Therefore we analyzed data both with and without OTUs considered as contamination by sequences at low levels in our negative controls, allowing us to observe the sex-specific differences in mice fed only a diet containing vitamin D.

A limitation of the current study was that the naïve mice did not receive a placebo treatment (i.e. Alum bolus and nebulisation). We also did not examine the microbiome of other locations including the BALF [15, 17], or the oro- [59] or naso-pharynx [60], which could limit comparisons with human studies. We could not discern cause and effect in our modeling: for example, with allergic airway disease did the induced inflammation change the microbiome (or vice versa)? A detailed time-course analysis and use of further control groups (i.e. Alum only) could help determine the cause and effect relationships in future studies.

Another consideration is the model of asthma used. This well-characterised model [61] involved sensitisation of mice with a ‘low dose’ of the allergen OVA (1 μg) with Alum (0.2 mg), which caused methacholine-induced airway hyperresponsiveness, airway eosinophilia and neutrophilia, increases in BALF levels of IL-5, and circulating allergen-specific IgE and IgG [24]. This model induces a phenotype similar to allergic asthma typified by a T helper type-2 (Th2) immune response, which is induced by a single well-defined allergen [61]. There is no ideal animal model of allergic asthma [62]; commonly known as allergic airway disease in mice. House dust mite (HDM) models offer an advantage of using a human allergen; however, HDM preparations can be contaminated with varying quantities of bacterial-derived lipopolysaccharide. The induction of neutrophilic and eosinophilic inflammation in HDM-induced allergic airway disease models are dependent on toll-like receptor-4 [63], complicating the interpretation of the inflammatory response [62], especially with regards to examining the lung microbiome. We have shown here that the induction of OVA-induced allergic airway disease significantly altered the lung microbiome and induced lung inflammation. We hypothesise that factors that modulate airway inflammation per se, including those induced by other allergens, such as OVA with Alum or HDM extract [61], will also modulate the lung microbiome to perpetuate pulmonary inflammation and disease.

In previous studies, we have shown that the effects of vitamin D deficiency on airway inflammation in male mice were dependent on the dose of OVA and Alum used to sensitise mice [24]. However, vitamin D deficiency did not significantly modify airway resistance, tissue elastance or damping in male mice with OVA-induced allergic airway disease induced by this low-dose sensitisation [24]. In other studies we examined the lung function of naïve vitamin D-deficient and -replete BALB/c mice and observed increased airway resistance and tissue damping in female (but not male) vitamin D-deficient but otherwise naive mice [43]. These observations suggest that any protective effect of vitamin D on lung function is limited to female mice, and combined with a trend for an inverse correlation between total OTUs in the lungs and serum 25(OH)D levels in female mice, may suggest that the lung microbiome could regulate lung function in a sex-dependent fashion. In addition, vitamin D may maintain optimal lung function by preventing airway remodeling through a process dependent on transforming growth factor-β [43].

In addition to the sex-dependent effects of vitamin D deficiency on the severity of lung inflammation in mice with allergic airway disease [24], we have previously shown that vitamin D deficiency increased the capacity of airway-draining lymph node cells from male and female mice to proliferate and produce Th2 cytokines [24, 25]. The effects of deficiency were reversed by subsequent supplementation with dietary vitamin D_3_ [24]. Vitamin D deficiency increased the influx of lymphocytes into BALF in response to exposure to HDM; however, deficiency was protective and reduced airway smooth muscle mass and airway resistance induced by HDM [65]. Increased OVA-specific IgE and IgG1 were detected in vitamin D-deficient female BALB/c mice following OVA/Alum sensitisation (without further respiratory challenge) [66]. Increased lung eosinophil numbers and CD4 + T1ST2+ cells (Th2 cells), and reduced CD4 + IL-10+ (regulatory) cells were observed in young adult offspring born to vitamin D-deficient BALB/c dams following chronic intranasal instillation of HDM to offspring from 3 days of age [67]. However, as observed in our studies with OVA-induced allergic airway disease, there was no effect of vitamin D deficiency on airway hyperresponsiveness [67]. Collectively, these studies suggest that vitamin D deficiency promotes Th2 responses and the accumulation of eosinophils and neutrophils in the lungs of mice with allergic airway disease, without further compromising lung function.

We detected a significant negative relationship between circulating 25(OH)D and total OTUs detected in the lungs, suggestive of reduced bacterial diversity with increasing serum 25(OH)D. Others have noted that bacterial diversity is increased in healthy lungs when compared to diseased lungs [23]. Patients with poorly controlled asthma (*n* = 30) had reduced bacterial diversity and species richness, with increased *Haemophilus influenzae* in sputum samples, particularly in younger males with increased neutrophils (*n* = 7) [23]. Changes in bacterial diversity were associated with oral corticosteroid intake, airway obstruction, and eosinophilia in lung lavage fluid [21]. Conversely, bacterial diversity in bronchial epithelial brushings was positively correlated with bronchial hyperresponsiveness of adults with sub-optimally controlled asthma (*n* = 65) [19]. However, those with increased baseline diversity had greater improvements in bronchial hyperresponsiveness in response to clathriomycin treatment [19].

## Conclusion

In these studies the capacity of OVA-induced allergic airway disease to modulate the lung microbiome and induce respiratory inflammation far exceeded any more subtle effects of vitamin D. We also show that the lung microbiome is differentially modified by sex in mice fed a vitamin D-containing diet, through specific effects on *Acinetobacter* OTU. It would be interesting to investigate the influence of other dietary or environmental modifiers on the lung microbiome, such as dietary fibre and dust from houses with dogs, which both change the gut microbiome, and have protective effects on the expression of allergic airway disease in mice [64, 68]. We identified a negative association between circulating 25(OH)D and OTUs of an unidentified *Pseudomonas*, which could be most clinically relevant for patients where *Pseudomonas* plays a central role in disease progression, such as for those with cystic fibrosis or COPD [69], and suggest that vitamin D status (or circulating 25(OH)D) may reduce the presence of pathobionts in the lungs. However, further studies in humans are required to reproduce the preclinical findings reported here.

## Abbreviations

1,25(OH)_2_D: 1,25-dihydroxyvitamin D
25(OH)D: 25-hydroxyvitamin D
Alum: Aluminium hydroxide suspension
ANOSIM: Analysis of similarity
BALF: Bronchoalveolar lavage fluid
COPD: Chronic obstructive pulmonary disease
HDM: House dust mite
mBD2: Mouse ß-defensin-2
NMDS: Non-metric multidimensional scaling
OTU: Operational taxonomic units
OVA: Ovalbumin
Th2: T helper type-2
